# Supplementary material for: Effectiveness and Safety of Ayurvedic Medicines in Type 2 Diabetes Mellitus Management: A Systematic Review and Meta-Analysis
Source: Front Pharmacol. 2022 Jun 8;13:821810. doi: 10.3389/fphar.2022.821810 (PMC9213670; doi:10.3389/fphar.2022.821810)
Supplement: Supplementary file 1 [file DataSheet1.zip › Supplementary Material/Forest and Funnel Plots/Punica granatum/TC.pdf]

Punica granatum - TC

Study	Intervention N	Intervention Mean	Intervention SD	Comparator N	Comparator Mean	Comparator SD	Mean Diff. with 95% CI	Weight (%)
Sohrab 2014, Sohrab 2015	22	136.10	48.60	22	189.90	58.20	-53.80 [-85.48, -22.12]	17.55
Faghihimani 2017	37	190.00	39.00	37	174.00	26.00	16.00 [0.90, 31.10]	23.16
Khajebishak 2019a, Khajebishak 2019b	26	171.70	30.80	26	154.30	76.30	17.40 [-14.23, 49.03]	17.57
Grabež 2020	19	194.10	51.04	18	209.18	28.61	-15.08 [-41.95, 11.79]	19.24
Hashemi 2020	30	159.62	33.52	30	165.42	34.97	-5.80 [-23.13, 11.53]	22.49
**Overall**							-6.88 [-30.59, 16.82]	

Heterogeneity: $\tau^2 = 572.29$, $I^2 = 81.49\%$, $H^2 = 5.40$

Test of $\theta_i = \theta_j$: $Q(4) = 18.18$, $p = 0.00$

Test of $\theta = 0$: $z = -0.57$, $p = 0.57$

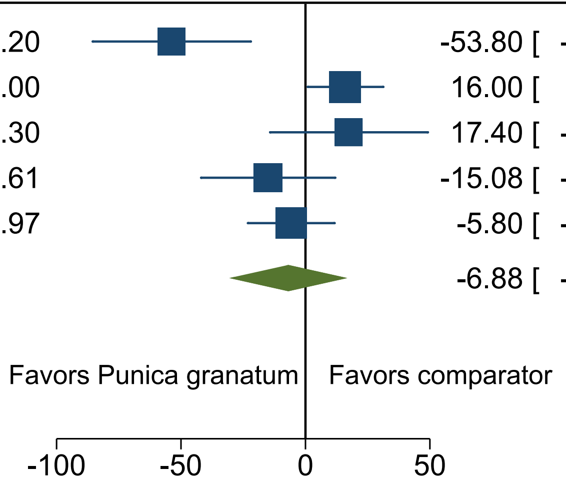

Random-effects REML model
